# European Society of Urogenital Radiology (ESUR) perspectives on the role of prostate MRI in active surveillance

**DOI:** 10.1186/s13244-026-02245-0

**Published:** 2026-04-02

**Authors:** Stephan Ursprung, Patricia A. Gutierrez, Ronaldo H. Baroni, Refky Nicola, Rafael Salvador, Pieter De Visschere, Derya Yakar, Francesco Giganti, Patrick Asbach

**Affiliations:** 1 Department of Diagnostic and Interventional Radiology, Tübingen University Hospital, Tübingen, Germany; 2 Radiology, The Netherlands Cancer Institute, Amsterdam, The Netherlands; 3 Department of Radiology, Centre Hospitalier Dunkerque, Dunkirk, France; 4 Department of Radiology, Hospital Israelita Albert Einstein, São Paulo, Brazil; 5 SUNY Upstate Medical University, Syracuse, NY USA; 6 Department of Radiology, Hospital Clínic de Barcelona, Universitat de Barcelona, Barcelona, Spain; 7 Department of Radiology and Nuclear Medicine, Ghent University Hospital, Ghent, Belgium; 8 Department of Radiology, University Medical Center Groningen, Groningen, The Netherlands; 9 Department of Radiology, University College London Hospital NHS Foundation Trust, London, UK; 10 Division of Surgery and Interventional Science, University College London, London, UK; 11 Department of Radiology, Charité - Universitätsmedizin Berlin, Corporate Member of Freie Universität Berlin and Humboldt-Universität zu Berlin, Campus Charité Mitte, Charitéplatz 1, Berlin, Germany

**Keywords:** Active surveillance, Magnetic resonance imaging, Prostate cancer

## Abstract

**Abstract:**

MRI has a central role in the diagnosis and management of prostate cancer, including active surveillance (AS) of low- and favourable intermediate-risk cancer. Robust evidence supports its use to guide biopsies and stratify the risk of progression at the inclusion stage. The Prostate Cancer Radiological Estimation of Change in Sequential Evaluation (PRECISE) criteria provide the foundation for standardised assessment on serial imaging during AS. Despite potential reductions in the number of unnecessary follow-up biopsies, uncertainty about the degree to which follow-up strategies can be defined by MRI leads to variation in international guidelines and their implementation. Here, the European Society of Urogenital Radiology (ESUR)–Prostate MRI Working Group reviews the evidence for the use of MRI in AS and provides practical guidance on its use. Additional research is needed to personalise AS strategies by integrating patient-specific factors, including family history and ethnicity, as well as emerging biomarkers such as genomic profiling and technological innovations like artificial intelligence.

**Critical relevance statement:**

MRI is an integral part of AS, and initiatives to standardise image acquisition and reporting are underway. Further research is needed to better define MRI’s role during follow-up and to personalise AS, which could help achieve better harmonisation among international guidelines. The European Society of Urogenital Radiology (ESUR) prostate working group provides suggestions for practical implementation.

**Patient summary:**

Active surveillance is a safe and effective management strategy for indolent prostate cancer. It avoids complications associated with surgery and radiation treatment. MRI has a central role in selecting which patients will benefit most from active surveillance and helping choose the most appropriate follow-up strategy. Acquiring standardised images and using reporting systems like PRECISE improves prostate cancer assessment and may help reduce the number of unnecessary biopsies.

**Key Points:**

- MRI plays a central role during active surveillance, but its implementation varies widely across centres.
- This ESUR position statement offers practical guidance on MRI acquisition, reporting, and interpretation tailored to active surveillance.
- Use of MRI—including the PRECISE score for active surveillance and the PI-QUAL score for image quality—can improve consistency and accuracy in monitoring prostate cancer over time.

**Graphical Abstract:**

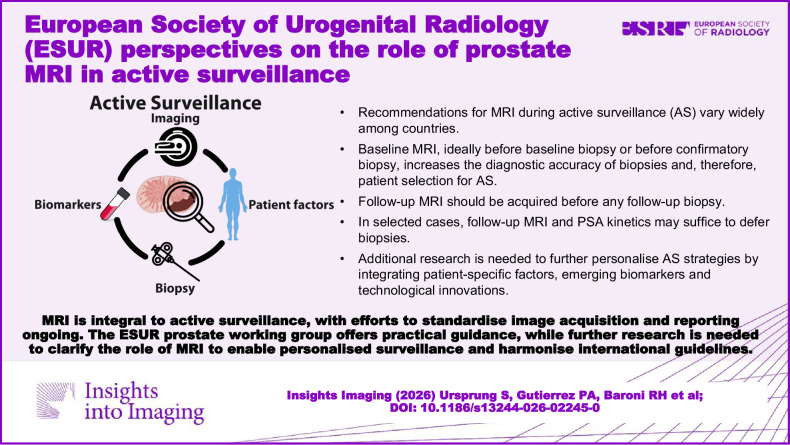

## Introduction

The introduction of the MRI-directed diagnostic pathway for prostate cancer (PCa) has achieved a significant reduction in the diagnosis of indolent disease compared to a biopsy pathway based solely on prostate-specific antigen (PSA) [1, 2]. The favourable benefit-to-harm ratios in major PCa screening trials exemplify the potential of MRI to reduce harm from overdiagnosis and overtreatment [3]. While MRI has a high sensitivity for clinically significant prostate cancer (csPCa) at the patient level, which is non-inferior to a systematic biopsy strategy, approximately 10% of csPCa, mostly smaller and lower-grade tumours, can be missed on MRI [4, 5]. Multiple strategies, including PSA-density (PSA-D) cutoffs, can help overcome this limitation [6].

Active surveillance (AS), the close monitoring of patients with low- or favourable intermediate-risk PCa, is supported by the disparity between the high prevalence of PCa and lower PCa mortality due to the heterogeneous and often slow-growing disease [7, 8]. Protocols for AS vary globally but commonly include PSA monitoring, digital rectal examination, imaging, and repeated biopsies. Level 1 evidence supports the use of MRI for identifying patients suitable for AS, demonstrating a reduction in failure rates and conversions to active treatment [9]. The evidence for using MRI during AS relies on cohort studies and is more controversial. While there may be advantages over per-protocol systematic biopsies and a potential to reduce the number of repeat biopsies [10], these benefits contrast findings that serial MRI alone is not accurate enough to determine progression or non-progression [11]. Prospective, randomised trials evaluating the benefit of MRI to detect cancer progression on AS are ongoing [12].

This special report, which is based on the unanimous agreement of members of the ESUR prostate working group, provides an overview of the role of MRI in AS from inclusion to follow-up and advises on the interpretation of MRI findings.

## “Indolent” prostate cancer—definition and controversy

AS aims to reduce unnecessary treatment and related side effects in patients whose cancer does not require immediate treatment [13]. The impact and success of the strategy, however, hinge on the inclusion criteria for AS as they are related to the concepts of indolent and clinically (non)significant prostate cancer. Clinical significance is attributed to cancers causing morbidity or mortality in a specific patient [13]. While initial definitions of csPCa were linked to radical prostatectomy (volume of index lesions > 0.5 mL, International Society of Urological Pathology (ISUP) grade group (GG) ≥ 2, and the presence of extraprostatic extension), criteria based on needle biopsy had to be established for AS [14, 15].

In the absence of universally accepted inclusion criteria, the major European stakeholders, including the European Association of Urology, the European Association of Nuclear Medicine, the European Society for Radiotherapy and Oncology, the European Association for Urogenital Radiology and the International Society of Geriatric Oncology, proposed joint recommendations to promote harmonisation [13]. These suggest offering AS to patients with a life expectancy exceeding 10 years, stage ≤ cT2b and a PSA < 10 ng/mL. Patients with a PSA 10–20 ng/mL may be enrolled in AS if the PSA-D is low. The exact threshold for this remains controversial. Patients with ISUP GG 2 tumours are only recommended to undergo AS if they fulfil additional, favourable criteria (PSA < 10 ng/mL, max. cT2a and low core positivity). Patients with high stage (cT2c+), large volume disease on MRI, intraductal growth, cribriform growth pattern, and ISUP GG ≥ 3 disease should not undergo AS [13]. However, as previously stated, recommendations and clinical practice vary among countries (Table 1).

**Table 1:** EAU, NCCN and country-specific criteria for inclusion in AS

Country	Source	Recommendation
Europe	European Association of Urology (EAU) prostate cancer guideline [59]	Inclusion: Low-risk: cT1c/cT2a, ISUP GG1, PSA < 10 ng/mL Intermediate-risk: GG2 with low G4 component < 10% Exclusion: ≥ cT3, cN+, PSA ≥ 15 ng/mL, cribriform, intraductal, ISUP GG ≥ 3
Australia	Cancer Council Australia—clinical practice guidelines for PSA testing and early management of test-detected prostate cancer [60]	PSA ≤ 20 ng/mL, ISPU GG1, ≤ cT2 Some patients with GG2 may be eligible
Brazil	[61]	There are no national guidelines. A 2022 survey reported that only 53% of urologists adopted AS for low- and very-low-risk PCa. Inclusion criteria were age > 50 years (32.2%), PSA < 10 ng/mL (87.2%), T1 clinical stage (80.4%), Biopsy Gleason score ≤ 6, positive cores ≤ 2 (44.3%), positive core involvement < 50% (45.3%), and magnetic resonance imaging findings (38.7%). PSA doubling time was used by 60.3%.
France	Association Française d’Urologie: Recommendations 2024–2026 [62]	Inclusion: Recommended for low risk: cT1c/cT2a, ISUP GG1, PSA < 10 ng/mL Consider for intermediate-risk: cT2b OR ISUP GG2 or PSA 10–20 ng/mL AND low tumour volume AND low percentage G4 AND low PSA-D
Germany	AWMF S3 Guideline version 8.1 2025 [27]	Inclusion: Low risk: cT1c/cT2a, ISUP GG1, PSA < 15 ng/mL Intermediate-risk: GG2 with Gleason pattern 4 < 10% Exclusion: ≥ cT3, cN+, PSA ≥ 15 ng/mL, cribriform, intraductal, ISUP GG ≥ 3
Japan	[63]	- ≤ cT2, PSA ≤ 20 ng/mL, PSA-D < 0.25 ng/mL^2^, ISUP GG1 (irrespective of number of positive cores) - GG2, < 50% positive cores (irrespective of the number of positive cores, if the patient underwent MRI fusion biopsy) - For MR-targeted biopsies, multiple positive cores at the target site are considered one positive core. - If saturation biopsy (≥ 20 cores) is performed, 15% of cores may be positive, with a maximum positivity rate of 4%.
Netherlands	PRIAS inclusion criteria [64]	Inclusion: - PSA ≤ 10 ng/mL, ≤ 20 ng/mL if MRI used - PSA density < 0.20 ng/mL^2^, if MRI negative or ISUP GG 1/2 < 0.25 ng/mL^2^ - cT1c or cT2 - ISUP GG 1 or 2 without intraductal or cribriform pattern - Limitation on the number of positive biopsy cores dependent on ISUP GG and MRI
South Africa	South African Prostate Cancer Guidelines—Draft Version 2017 [65]	- PSA ≤ 10 ng/mL, ISPU GG1, ≤ cT2 - GG2 with small percentage pattern 4
South Korea	[66]	- PSA ≤ 20 ng/mL, ISPU GG1–2, ≤ cT2 - No cribriform pattern - Positive core ratio ≤ 50% - Cancer involvement ≤ 50%/core
United Kingdom	National Institute for Health and Care Excellence (NICE) guideline 2021 [67]	Offer: Cambridge Prognostic Group 1 and 2: cT1/2 AND ISUP GG 1 AND PSA ≤ 20 ng/mL OR cT1/2 AND ISUP GG 2 AND PSA ≤ 10 ng/mL Consider: Cambridge Prognostic Group 3: cT1/2 AND ISUP GG 2 AND PSA 10–20 ng/mL OR cT1/2 AND ISUP GG 3 Do not offer: Cambridge Prognostic Group 4–5: any of: ≥ cT3, ISUP GG 4/5, PSA > 20 ng/mL
USA	National Comprehensive Cancer Network (NCCN) guideline 1.2026 [68]	Inclusion: (Very) low risk: cT1/cT2a, ISUP GG 1, PSA < 10 ng/mL Favourable intermediate risk: one risk factor of cT2b/cT2c, ISUP GG 2, PSA 10–20 ng/mL AND < 50% biopsy cores positive Exclusion: cT3–cT4, ISUP GG 4 or 5, PSA > 20 ng/mL, unfavourable histology

Some of the consensus criteria enjoy broad consensus; for example, ISUP GG, tumour stage, and serum PSA are major predictors of adverse outcomes and, therefore, clinical significance of PCa [16]. However, tumour grading has evolved over time as adaptations to the definition of PCa grades have led to grade inflation [17]. The increasing use of MRI-guided biopsies in addition to or instead of systematic biopsies allows targeting the most aggressive part of tumours, contributing to an increase in histological tumour grade [18]. As a result, today’s ISUP GG 2 (Gleason 3 + 4) cancers have a better prognosis than historical Gleason 3 + 4 tumours [19].

The willingness to include patients with ISUP GG 2 tumours in AS has been increasing. AS is a safer treatment option for selected patients with favourable intermediate-risk PCa [20]. However, the risk of transitioning to active treatment is increased compared to patients with ISUP GG 1 tumours [20]. Consequently, routine use of AS in patients with favourable intermediate-risk tumours is still controversial [21–23]. Prostate MRI may contribute to an improved selection of intermediate-risk patients for AS, as visibility of ISUP GG 2 lesions is associated with a switch to active treatment [24].

The number of positive cores and the percentage of tumour within a core in systematic biopsies have been proposed as further selection criteria for AS and entered in some clinical guidelines (e.g., the NCCN guidelines on prostate cancer 1.2026) [25]. However, there is little basis for excluding patients from AS based on core features that are either not or only weakly associated with adverse findings at prostatectomy [26]. Furthermore, evolving biopsy strategies and the increasing use of MR-guided biopsies and perilesional sampling have rendered biopsy criteria less applicable as a surrogate for lesion size and risk [14]. Consequently, other guidelines, including the German S3 guidelines, have abandoned these criteria [27].

The evidence suggests that patient characteristics, in addition to disease features, influence the success of an AS strategy. A recent meta-analysis showed that the black population on AS is at an increased risk of requiring treatment or experiencing clinical progression [28]. In contrast, the Asian population may experience progression less frequently than the Caucasian population. However, they may convert to active treatment in the absence of progression more frequently, which may be due to differences in institutional protocols and the perception of what constitutes acceptable risk [29]. Taking into account patient characteristics when defining AS eligibility and follow-up is central to realising its full potential.

## MRI of the prostate before enrolment of patients into active surveillance

Although mpMRI has become a cornerstone in the diagnostic pathway of PCa, it is not yet uniformly integrated into the inclusion criteria for AS across Europe. In many centres, systemic biopsy results alone determine eligibility, and MRI follows (incidental) diagnosis, such as through transurethral resection of the prostate or biopsy [30, 31]. National guidelines and clinical practice patterns throughout Europe reflect this variability (Table 1).

The use of MRI prior to confirmatory biopsy improves diagnostic accuracy by enabling lesion-targeted sampling. The positive predictive value of a Prostate Imaging Reporting and Data System (PI-RADS) 4–5 lesion ranges between 40% and 70%, depending on PSA-D and biopsy approach [25]. When MRI findings are negative (PI-RADS ≤ 2) and PSA-D is < 0.15 ng/mL/cm³, the negative predictive value for ruling out csPCa can exceed 90%, supporting its role in safely deferring invasive procedures in carefully-selected patients [25]. In the ProtecT cohort, which did not include MRI in its original design, retrospective analysis suggests that 30–50% of patients may have been reclassified with MRI-targeted biopsy [32]. This agrees with studies showing that 20–30% of patients initially considered eligible for AS based on systematic biopsy are reclassified when MRI-targeted biopsies are performed [25]. Crucial evidence supports the safety and efficiency of AS in the era of MRI-directed PCa diagnosis, underpinned by the high negative predictive value of prostate MRI [33, 34]. Nonetheless, the optimal management of patients with negative MRI who are at a high risk of prostate cancer needs additional investigation [35].

These findings support MRI as a stratification tool, particularly in light of emerging data showing significant genomic differences between lesions sampled via MRI-targeted versus systematic biopsy [36], which may impact long-term outcomes and eligibility for AS. Despite growing evidence, MRI implementation remains inconsistent. International consensus efforts, such as the Delphi process led by Bruinsma et al, have highlighted the persistent heterogeneity in terminology, risk definitions, and diagnostic workup prior to AS [37]. Cultural and practice-based differences, including physician attitudes and patient preferences, further shape MRI utilisation, as shown in recent evaluations of decision-making behaviour [38, 39]. These findings underscore the need for standardised, MRI-informed pathways to ensure appropriate selection and long-term safety in AS.

## MRI of the prostate during active surveillance

Adherence to AS varies considerably between countries and among centres in single countries [40, 41]. While adherence is particularly high in Nordic countries (e.g., in Sweden, 88% of patients diagnosed with low-risk PCa between 2020 and 2024 chose AS [42]), the latest available data from Germany show only 27% of eligible patients opt for AS [43]. The aim of AS is to differentiate the approximately 27% of patients experiencing disease progression from those who can safely continue AS [11, 44]. Follow-up biopsies are key to identifying patients whose tumour was undergraded owing to sampling error at baseline or progressed. Nonetheless, biopsies are underutilised [45]. Meta-analyses found that MRI alone is insufficiently accurate to supplant follow-up biopsies [11, 44]. However, its combination with other biomarkers may reduce the number of re-biopsies, making AS less onerous for patients [41]. Recommendations on the use and implications of serial MRI during AS vary, reflecting a paucity of evidence (Table 2).

**Table 2:** EAU and country-specific recommendations on MRI during follow-up

Country	MRI recommendations	Biopsy recommendations	Reference
Europe/EAU	The AS strategy should be based on PSA (at least once every 6 months), serial DRE (at least once yearly) and repeated biopsy. Serial DRE may be omitted if MRI is stable. A stable MRI (PRECISE 1–3) does not make repeat biopsy superfluous, but in patients with low-risk tumour and a stable low PSA-D < 0.15 may be excluded.	Perform per-protocol confirmatory prostate biopsies if MRI is not available. Repeated biopsy (every 2–3 years for 10 years). Perform MRI and repeat biopsy if PSA is rising (PSA-doubling time < 3 years).	EAU Prostate Cancer Guideline 2024 [59]
Australia		Offer a reclassification repeat prostate biopsy within 6–12 months of starting an AS protocol. Further repeat biopsies every 2–3 years or sooner if prompted by clinical findings.	Cancer Council Australia—clinical practice guidelines for PSA testing and early management of test-detected prostate cancer [60]
Brazil		No national guidelines. A 2022 survey reported that confirmatory biopsy (55.9%), PSA level (36.6%), and digital rectal examination (34.4%) are used in follow-ups. Patient preference (85.7%), upgrade of Gleason score (73.4%), and increased number of positive cores (66.8%) were associated with conversion to definitive treatment.	[61]
France	MRI preceding control biopsies. A normal control MRI does not make it possible to avoid biopsies.	First control biopsy after 6–18 months. Subsequent biopsies are risk-adapted.	French AFU Cancer Committee [62]
Germany	If no baseline MRI, MRI within 6 months of starting AS + targeted biopsy of target lesions ≥ PI-RADS 3. If baseline MRI is performed, repeat MRI after 12–18 months on AS	Repeat targeted and systematic biopsy after repeat MRI at 12–18 months. Further repeat biopsies as a function of MRI and PSA results, but at least every 3 years during the first 10 years of AS.	AWMF S3-Leitlinie Prostatakarzinom [27]
Japan	None	Biopsy at 1 year, 3-yearly until year 10, 5-yearly thereafter 3-monthly PSA for 2 years, 6-monthly thereafter DRE 6-monthly	[63]
Netherlands	If no baseline MRI, MRI within 3 months of starting AS. Repeat MRI at 1, 4, 7 and 10 years. Subsequently, 5-yearly. If PSA-doubling time < 10 years, annual MRI.	Repeat biopsies after repeat MRI at 1, 4, 7 and 10 years. Subsequently, 5-yearly. If PSA-doubling time < 10 years, biopsy of new or progressive lesions on annual MRI.	PRIAS project [64]
South Africa	None	- Regular follow-up with DRE and PSA - Biopsy every 12–24 months on clinical indication - PSA-D < 0.15–0.2 ng/mL^2^ - ≤ 50% PCa/core	South African Prostate Cancer Guidelines—Draft Version 2017 [65]
South Korea	Annually	Repeat biopsy after 1 year, thereafter at the physician’s discretion 6-monthly PSA	[66]
United Kingdom	mpMRI is used at 12 months post-diagnosis and subsequently every 3–5 years. MRI findings guide the necessity of repeat biopsies.	Repeat biopsies are considered if MRI indicates progression or if PSA levels rise.	National Institute for Health and Care Excellence (NICE) Guideline 2021 [67]
USA	Serial PSA increases, new DRE abnormalities, or other concerns for clinical progression prompt re-evaluation with MRI and possible prostate biopsy. In patients selecting AS, clinicians should utilise mpMRI to augment risk stratification, but this should not replace periodic surveillance biopsy.	PSA values, obtained no more frequently than 6-monthly, symptom re-assessment and physical examination with DRE every 1 to 2 years. If the mpMRI shows PI-RADS 1, 2, or 3, repeat biopsy may be performed within 12 months after diagnosis. Thereafter, serial surveillance biopsies every 1 to 4 years, depending on patient age, health, risk of progression, and preference.	AUA/ASTRO Guidelines [69]

Standardised image acquisition and reporting are equally relevant in the diagnostic and AS setting. Therefore, the Prostate Cancer Radiological Estimation of Change in Sequential Evaluation (PRECISE) panel developed expert recommendations on conducting and interpreting serial MRI in 2016 [46], and these recommendations have been recently updated (PRECISE v2) [47].

**Fig. 1:**
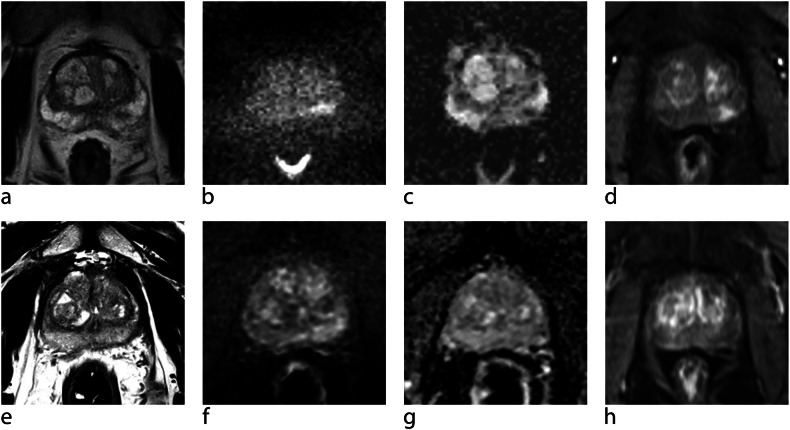
An approx 65-year-old man with elevated PSA who had undergone MRI-informed targeted biopsy for a PI-RADS 4 lesion in the left peripheral zone. **a** Axial T2-TSE, (**b**) calculated b1400 s/mm^2^ image, (**c**) ADC-map, (**d**) DCE image. Histology revealed ISUP 1 prostate cancer in 2/12 cores (left peripheral zone) and active inflammation. On follow-up MRI after 16 months, the lesion has reduced in size and conspicuity (PRECISE 2). **e** Axial T2-TSE, (**f**) calculated b1400 s/mm^2^ image, (**g**) ADC-map, (**h**) DCE image

**Fig. 2:**
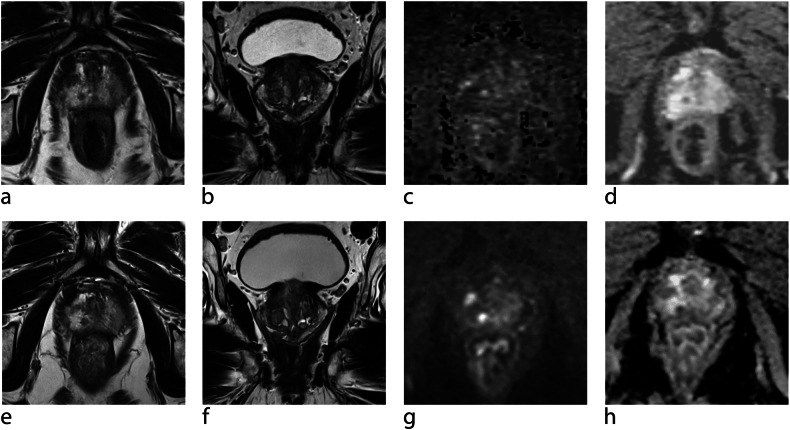
An approx 70-year-old man with elevated PSA who had undergone MRI-informed targeted biopsy for a PI-RADS 4 lesion in the right peripheral zone at the apex. **a** Axial T2-TSE, (**b**) coronal T2-TSE, (**c**) calculated b1400 s/mm^2^ image, (**d**) ADC-map. Histology revealed ISUP 1 prostate cancer in 3/12 cores (right peripheral zone). On follow-up MRI after 24 months, the lesion has not changed in size and conspicuity; what appears to be more focal marked diffusion restriction is related to a more advanced diffusion technique using a different MR scanner (PRECISE 3-V). **e** Axial T2-TSE, (**f**) coronal T2-TSE, (**g**) calculated b1400 s/mm^2^ image, (**h**) ADC-map

The PRECISE score evaluates the degree of radiological change over time. A PRECISE score of 1 indicates complete resolution of previously equivocal/suspicious findings, while a PRECISE score of 5 indicates definite radiologic stage progression. In PRECISE v2, there is also an additional category of PRECISE X for single non-diagnostic studies of poor image quality (Table 3) [47] as image quality should be evaluated for each single scan (e.g., PI-QUAL v2; [48]). If the quality of a baseline MRI is insufficient, a good-quality second scan may serve as a baseline for PRECISE assessment. For PRECISE scoring, follow-up scans shall be compared to the baseline scan and the immediately preceding scan (Figs. 1 and 2). The appearance of new lesions > 0.2 cm^2^ (6 mm diameter), new focal lesions (PI-RADS/Likert ≥ 3), a volume increase > 50%, an increase in conspicuity, or stage progression constitutes significant progression. Lesion size shall be measured with at least two diameters.

**Table 3:** PRECISE v2 scoring system, adapted from [47]

PRECISE score	Likelihood of radiological change	Examples
1	Complete resolution of suspicious features	Disappearance of focal lesion on all sequences
2	Reduction in size and/or conspicuity of suspicious areas	Disappearance of a peripheral zone lesion on high b-value images with maintained low signal on ADC maps (PI-RADS 4 -> PI-RADS 3)
3 non-visible	Stable: no visible lesion	Stable diffuse changes
3 visible	Stable visible lesion(s)	Stable size and conspicuity of lesions
4	Significant increase in size and/or conspicuity of suspicious area; new focal lesion	> 50% increase in volume or appearance on new sequences, e.g., peripheral zone lesion PI-RADS 3 -> PI-RADS 4
5	Definite radiological stage progression	Suspicion of extracapsular extension (T3a), seminal vesicle invasion (T3b) or nodal/distant metastasis (N+, M+)
X	PRECISE scoring impossible	Suboptimal image quality

Owing to its recent update, no cohort study on PRECISE v2 has been published yet. Of note, the most recent meta-analyses, including publications until 03/2020 [44] and 01/2021 [11], found that MRI follow-up was not sufficiently accurate to alter management without biopsy. However, its high negative predictive value of 0.81–0.85 may help reduce the number of biopsies. The meta-analysis by Rajwa et al observed a nonsignificant trend toward improved diagnostic estimates of PRECISE recommendations compared to institution-specific criteria. Recent publications on PRECISE v1 reported performances comparable to the meta-analyses [49, 50]. When interpreting the sensitivity of MRI for grade progression, upgrading rates of confirmatory biopsies need to be considered. In the MRI era, still 10–20% of patients are upgraded at confirmatory biopsy, suggesting undersampling at baseline rather than true grade progression [51].

In PRECISE v2, radiological stability (i.e., PRECISE 3) is further stratified into patients with visible (3 V) and non-visible (3Non-V) disease. Patients without MR-visible lesions have a lower risk of upgrading on follow-up biopsies, transitioning to active treatment and death. Low-risk disease without visible lesions has an excellent prognosis [52]. The low-risk subgroup of low-risk PCa without lesions on MRI and stable PSA presents an opportunity to reduce follow-up biopsies [24].

## ESUR perspectives on the use of MRI in active surveillance

**Fig. 3:**
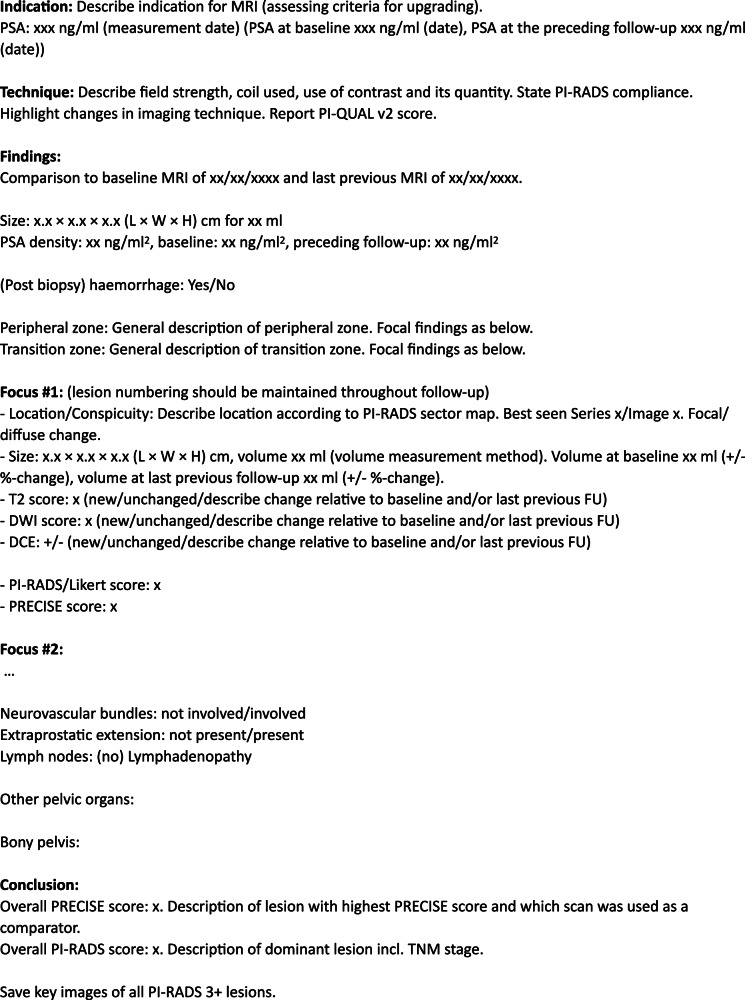
Report template for prostate MRI obtained during active surveillance, including PI-QUAL, PI-RADS and PRECISE scoring. For an example of a visual report, please refer to [47]

Despite increasing adoption of MRI in active surveillance (AS), technical and interpretative variability persists in clinical practice. To promote standardisation, we defined ESUR perspectives regarding image acquisition, quality, reporting and longitudinal interpretation in AS, based on their experience with AS (Supplementary Table 1). These core concepts—summarised in Table 4—are intended to assist radiologists and urologists in delivering high-quality, reproducible MRI-based follow-up. Figure 3 includes a template for reporting prostate MRI for AS. A dedicated PRECISE v2 case report form is available and particularly recommended for use in research contexts [47].

**Table 4:** ESUR perspectives for MRI in active surveillance

Technical aspects	1. MRI acquisition should follow the PI-RADS v2.1 guidelines. Acquire images in straight axial/coronal/sagittal orientation and avoid angulation to improve the comparability between baseline and repeat MRI.
	2. Image quality must be assessed with a standardised scoring system (e.g., PI-QUAL). Multiparametric scans with PI-QUAL (v2) scores of 2 and 3 are suitable for PRECISE scoring (preference PI-QUAL score 3). Low-quality/non-diagnostic quality images (PI-QUAL score 1) should not be interpreted for AS (i.e., PRECISE X).
	3. MRI without contrast medium can be considered for AS in the absence of biochemical progression, if image quality is good (PI-QUAL score 3).
Timing	4. Baseline MRI is required before enrolling patients into AS and should ideally be acquired before baseline biopsy or within 6 months (e.g., if PCa was diagnosed during TURP).
	5. Repeat MRI during AS should be performed after at least 12 months. Follow-up biopsies during AS should be preceded by MRI. The follow-up strategy should be risk-adapted and may trigger MRI after 12–36 months.
Interpretation and reporting	6. A dedicated standardised scoring system should be used for image interpretation. The ESUR recommends the PRECISE score be used (Table 3 and Figs. 1–3, Supplementary Figs. 1 and 2). 7. The radiologist should be experienced in reporting prostate MRI.
	8. Changes in the appearance of the disease should be classified relative to the baseline scan/the first available scan, and to the immediately preceding scan. 9. The comparison to the baseline scan overrules the comparison with the more recent scans in case of progression (i.e., overall PRECISE score).
	10. The differentiation between stable, visible and stable, non-visible lesions (PRECISE 3-V/Non-V) is crucial for predicting the risk of progression and should always be reported.
	11. Size/volume of lesions is an important factor in monitoring the disease over time. However, no standardised method for volumetric measurement is widely available. Therefore, the cutoff for progression (50% volume increase according to PRECISE) may be difficult to evaluate—the same method (e.g., planimetry) should be used for all scans.
	11. Differences on follow-up imaging may also be related to technical variations between scanners or vendors (i.e., they might mimic disease progression), and this should be mentioned in the report to avoid confusion with true progression (Fig. 2)
	12. A PI-RADS or Likert score should be reported at each follow-up time point.
	13. Pictorial lesion mapping is an integral part of the MRI report (e.g., sector map, screenshot, PRECISE case report form).

*AS* Active Surveillance, *ESUR* European Society of Urogenital Radiology, *MRI* Magnetic Resonance Imaging, *PI-QUAL* Prostate Imaging Quality, *PI-RADS* Prostate Imaging Reporting And Data System, *PRECISE* Prostate Cancer Radiological Estimation of Change In Sequential Evaluation, *TURP* TransUrethral Resection of the Prostate

## Future perspectives

The success of AS hinges on oncological safety, acceptability of follow-up protocols to patients, and clear pathways for inclusion, follow-up, and intervention. Notwithstanding the increasing agreement on the inclusion criteria for AS (low-risk and favourable intermediate-risk PCa), the failure rate of AS remains considerable, with approximately 30% of patients requiring active treatment at 5 years [52]. That highlights the potential for further improvements in patient stratification. While its value is still uncertain, additional biomarkers such as tumour genomic profiles may supplement baseline MRI, biopsy and PSA to better characterise PCa progression risk and help identify patients most suitable for AS [53].

Improved personalisation of AS strategies will also require adaptation to individual risk factors. Patients in the Black community are at an increased risk of developing PCa and experiencing disease progression [54]. Simultaneously, they are less able to access diagnostic MRI as part of AS [55]. Similarly, Asian patients are at an increased risk of advanced disease at presentation. Consequently, patients considered suitable for AS according to criteria developed in predominantly Caucasian populations may be at an increased risk of undergrading and understaging. This suggests that a one-size-fits-all approach to inclusion in AS and surveillance protocols compromises the oncological safety and efficacy of AS. Consequently, research in more diverse populations is essential, and guidelines need to take additional patient factors into account.

Variations in the role of MRI during follow-up on AS across European guidelines mirror its rapid evolution. Future prospective research will determine to what degree a combination strategy of imaging and biomarker follow-up may help reduce the frequency of protocol biopsies. Furthermore, future research needs to define how follow-up schedules can adapt to the risk of progression and how repeat imaging is triggered to achieve a high sensitivity for progressive PCa with an acceptable specificity. Standardisation in image acquisition and interpretation is of increased importance in AS, particularly as patients may undergo imaging on various equipment and in different centres. While lesions with a decreasing apparent diffusion coefficient may appear more conspicuous (PRECISE 4), quantitative maps obtained from diffusion-weighted or dynamic contrast-enhanced imaging vary considerably between protocols [56]. Furthermore, if MRI is intended to partly replace invasive biopsies, the clinical significance of progressing lesions that are undetected by MRI needs to be investigated [57].

Recent research highlighted the potential of artificial intelligence to address some of the unmet clinical needs in AS, including improved patient selection and personalising the intensity of follow-up examinations by predicting progression to csPCa [58].

In conclusion, this report, prepared on behalf of the entire ESUR Prostate Working Group, provides practical guidance on the role of prostate MRI during active surveillance, emphasising the importance of the PRECISE v2 recommendations, while recognising that further work is required to enable personalised surveillance and harmonise international guidelines.

## Supplementary information

ELECTRONIC SUPPLEMENTARY MATERIAL

Supplementary information

Supplementary information

## Data Availability

No original data were produced as part of this work.

## Abbreviations

AS: Active surveillance
(cs)PCa: (Clinically significant) prostate cancer
EAU: European Association of Urology
ESUR: European Society of Urogenital Radiology
GG: Grade group
ISUP: International Society of Urological Pathology
mpMRI: Multiparametric magnetic resonance imaging
NCCN: National Comprehensive Cancer Network
NICE: National Institute for Health and Care Excellence
PI-QUAL: Prostate imaging quality
PI-RADS: Prostate Imaging Reporting and Data System
PRECISE: Prostate cancer radiological estimation of change in sequential evaluation
PSA(-D): Prostate-specific antigen (density)
