# Supplementary material for: European Society of Urogenital Radiology (ESUR) perspectives on the role of prostate MRI in active surveillance
Source: Insights Imaging. 2026 Apr 2;17:87. doi: 10.1186/s13244-026-02245-0 (PMC13046944; doi:10.1186/s13244-026-02245-0)
Supplement: Supplementary file 1 — ELECTRONIC SUPPLEMENTARY MATERIAL [file 13244_2026_2245_MOESM1_ESM.pdf]

European Society of Urogenital Radiology (ESUR) Perspectives on the Role of Prostate MRI in Active Surveillance

ELECTRONIC SUPPLEMENTARY MATERIAL

Supplementary Figure S1:

Clinical History: Date of PCa diagnosis: November 2023. PSA: 8 ng/ml (March 2025) (PSA at baseline 9.5 ng/ml (October 2023).
Indication: MRI during active surveillance: assessing criteria for upgrading.

Technique: 3 Tesla MRI with phased-array surface coils. Localizer scan, axial T1w-GRE and sagittal T2w-TSE of the entire pelvis. High spatial resolution axial and coronal T2w-TSE-sequences and DWI of the prostate. DCE (VIBE sequence) after i.v. administration of 14 ml GBCA. PI-QUAL v2.: 3.

Findings:
Comparison to baseline MRI from October 2023.

Size: 48 × 46 × 45 (L × W × H) cm - volume 45 ml
PSA density: 0.18 (ng/ml)/cm^3, baseline: 0.21 (ng/ml)/cm^3

(Post biopsy) haemorrhage: absent.

Peripheral zone: mild linear T2-hypointensities, specifically in the left lateral peripheral zone (site of prior focal lesion).
Transition zone: typical findings compatible with BPH.

Focus #1:
- Mild linear to wedge shaped changes left peripheral zone, no focal lesion (L1, Series 12, Ima 14, PZpl).
- T2 score: 2 (prior focal lesion has partially resolved with some residual linear changes)
- DWI score: 2 (prior focal lesion has partially resolved)
- DCE: - (prior focal lesion has partially resolved, no focal enhancement in the current scan)

- PI-RADS/Likert score: 2
- PRECISE score: 2

Focus #2:
Extraprostatic extension: very unlikely
Seminal vesicles: not involved
Lymph nodes: no lymphadenopathy

Other pelvic organs: unremarkable

Bony pelvis: unremarkable

Conclusion:
PRECISE score: 2: prior focal lesion in the left peripheral zone has partially resolved with some residual linear changes (L1, PI-RADS 2). No criteria for upgrading to clinically significant disease. No evidence for EPE or pelvic metastatic disease.

Supplementary Figure S2:
Clinical History: Date of PCa diagnosis: July 2022. PSA: 5.5 ng/ml (June 2024) (PSA at baseline 4.5 ng/ml (June 2022).
Indication: MRI during active surveillance: assessing criteria for upgrading.

Technique: 3 Tesla MRI with phased-array surface coils. Localizer scan, axial T1w-GRE and sagittal T2w-TSE of the entire pelvis. High spatial resolution axial and coronal T2w-TSE-sequences and DWI of the prostate.
PI-QUAL v2.: 3.

Findings:
Comparison to baseline MRI from June 2024.

Size: 44 × 42 × 43 (L × W × H) cm - volume 41 ml
PSA density: 0.13 (ng/ml)/cm^3, baseline: 11 (ng/ml)/cm^3

(Post biopsy) haemorrhage: absent

Peripheral zone: two focal lesions at the right apex, otherwise homogeneous high T2 signal intensity.
Transition zone: typical findings compatible with BPH.

Focus #1:
- 5x5x4 mm lesion right anterolateral peripheral zone (L1, Series 12, Ima 17, PZa).
- T2 score: 4 (unchanged)
- DWI score: 4 (unchanged, what appears to be more focal marked diffusion restriction is related to a more advanced diffusion technique)
- DCE: x

- PI-RADS/Likert score: 4
- PRECISE score: 3-V

Focus #2:
- 3x3x2 mm lesion right posterior peripheral zone (L1, Series 12, Ima 18, PZpm).
- T2 score: 4 (unchanged)
- DWI score: 4 (unchanged / what appears to be more focal marked diffusion restriction is related to a more advanced diffusion technique)
- DCE: x

- PI-RADS/Likert score: 4
- PRECISE score: 3-V

Focus #3:
Extraprostatic extension: very unlikely
Seminal vesicles: not involved
Lymph nodes: no lymphadenopathy

Other pelvic organs: unremarkable

Bony pelvis: unremarkable

Conclusion:
Overall PRECISE score: 3-V. The two focal lesions in the right peripheral zone at the apex (L1 and L2, PI-RADS 4) are stable and compatible with the diagnosis of GG1 prostate cancer. No criteria for upgrading to clinically significant disease.
No evidence for EPE or pelvic metastatic disease.

Supplementary Table S1. *Experience of the 9 authors with active surveillance in prostate cancer*

Number of AS patients reported per year	< 50<br>n=1	50-100<br>n=4	> 100<br>n=4
Experience in reporting Active Surveillance	< 2 years<br>n=0	2-5 years<br>n=3	> 5 years<br>n=6
Reporting AS routinely includes the PRECISE score	yes<br>n=8	no<br>n=1	
Reporting AS also includes patients with Gleason 3+4=7a (GG2) disease	yes<br>n=1	no<br>n=8	
