# Supplementary material for: European Society of Urogenital Radiology (ESUR) perspectives on the role of prostate MRI in active surveillance
Source: Insights Imaging. 2026 Apr 2;17:87. doi: 10.1186/s13244-026-02245-0 (PMC13046944; doi:10.1186/s13244-026-02245-0)
Supplement: Supplementary file 2 — Supplementary information [file 13244_2026_2245_MOESM2_ESM.pdf]

Clinical History: Date of PCa diagnosis: November 2023. PSA: 8 ng/ml (March 2025) (PSA at baseline 9.5 ng/ml (October 2023).
Indication: MRI during active surveillance: assessing criteria for upgrading.

Technique: 3 Tesla MRI with phased-array surface coils. Localizer scan, axial T1w-GRE and sagittal T2w-TSE of the entire pelvis. High spatial resolution axial and coronal T2w-TSE-sequences and DWI of the prostate. DCE (VIBE sequence) after i.v. administration of 14 ml GBCA. PI-QUAL v2.: 3.

Findings:
Comparison to baseline MRI from October 2023.

Size: 48 × 46 × 45 (L × W × H) cm - volume 45 ml
PSA density: 0.18 (ng/ml)/cm^3, baseline: 0.21 (ng/ml)/cm^3

(Post biopsy) haemorrhage: absent.

Peripheral zone: mild linear T2-hypointensities, specifically in the left lateral peripheral zone (site of prior focal lesion).
Transition zone: typical findings compatible with BPH.

Focus #1:
- Mild linear to wedge shaped changes left peripheral zone, no focal lesion (L1, Series 12, Ima 14, PZpl).
- T2 score: 2 (prior focal lesion has partially resolved with some residual linear changes)
- DWI score: 2 (prior focal lesion has partially resolved)
- DCE: - (prior focal lesion has partially resolved, no focal enhancement in the current scan)

- PI-RADS/Likert score: 2
- PRECISE score: 2

Focus #2:
Extraprostatic extension: very unlikely
Seminal vesicles: not involved
Lymph nodes: no lymphadenopathy

Other pelvic organs: unremarkable

Bony pelvis: unremarkable

Conclusion:
PRECISE score: 2: prior focal lesion in the left peripheral zone has partially resolved with some residual linear changes (L1, PI-RADS 2). No criteria for upgrading to clinically significant disease.
No evidence for EPE or pelvic metastatic disease.
