# Supplementary material for: European Society of Urogenital Radiology (ESUR) perspectives on the role of prostate MRI in active surveillance
Source: Insights Imaging. 2026 Apr 2;17:87. doi: 10.1186/s13244-026-02245-0 (PMC13046944; doi:10.1186/s13244-026-02245-0)
Supplement: Supplementary file 3 — Supplementary information [file 13244_2026_2245_MOESM3_ESM.pdf]

Clinical History: Date of PCa diagnosis: July 2022. PSA: 5.5 ng/ml (June 2024) (PSA at baseline 4.5 ng/ml (June 2022).
Indication: MRI during active surveillance: assessing criteria for upgrading.

Technique: 3 Tesla MRI with phased-array surface coils. Localizer scan, axial T1w-GRE and sagittal T2w-TSE of the entire pelvis. High spatial resolution axial and coronal T2w-TSE-sequences and DWI of the prostate.
PI-QUAL v2.: 3.

Findings:
Comparison to baseline MRI from June 2024.

Size: 44 × 42 × 43 (L × W × H) cm - volume 41 ml
PSA density: 0.13 (ng/ml)/cm^3, baseline: 11 (ng/ml)/cm^3

(Post biopsy) haemorrhage: absent

Peripheral zone: two focal lesions at the right apex, otherwise homogeneous high T2 signal intensity.
Transition zone: typical findings compatible with BPH.

Focus #1:
- 5x5x4 mm lesion right anterolateral peripheral zone (L1, Series 12, Ima 17, PZa).
- T2 score: 4 (unchanged)
- DWI score: 4 (unchanged, what appears to be more focal marked diffusion restriction is related to a more advanced diffusion technique)
- DCE: x

- PI-RADS/Likert score: 4
- PRECISE score: 3-V

Focus #2:
- 3x3x2 mm lesion right posterior peripheral zone (L1, Series 12, Ima 18, PZpm).
- T2 score: 4 (unchanged)
- DWI score: 4 (unchanged / what appears to be more focal marked diffusion restriction is related to a more advanced diffusion technique)
- DCE: x

- PI-RADS/Likert score: 4
- PRECISE score: 3-V

Focus #3:
Extraprostatic extension: very unlikely
Seminal vesicles: not involved
Lymph nodes: no lymphadenopathy

Other pelvic organs: unremarkable

Bony pelvis: unremarkable

Conclusion:
Overall PRECISE score: 3-V. The two focal lesions in the right peripheral zone at the apex (L1 and L2, PI-RADS 4) are stable and compatible with the diagnosis of GG1 prostate cancer. No criteria for upgrading to clinically significant disease.
No evidence for EPE or pelvic metastatic disease.
